# Camel Grass Phenolic Compounds: Targeting Inflammation and Neurologically Related Conditions

**DOI:** 10.3390/molecules27227707

**Published:** 2022-11-09

**Authors:** Graciliana Lopes, Elisabete Gomes, Mariana Barbosa, João Bernardo, Patrícia Valentão

**Affiliations:** REQUIMTE/LAQV, Laboratory of Pharmacognosy, Department of Chemistry, Faculty of Pharmacy, University of Porto, Rua de Jorge Viterbo Ferreira, n° 228, 4050-313 Porto, Portugal

**Keywords:** *Cymbopogon schoenanthus*, hyaluronidase, acetylcholinesterase, neurodegeneration, oxidative stress, phenolic compounds

## Abstract

Background: The use of plants for therapeutic purposes has been supported by growing scientific evidence. Methods: This work consisted of (i) characterizing the phenolic compounds present in both aqueous and hydroethanol (1:1, *v*/*v*) extracts of camel grass, by hyphenated liquid chromatographic techniques, (ii) evaluating their anti-inflammatory, antioxidant, and neuromodulation potential, through *in vitro* cell and cell-free models, and (iii) establishing a relationship between the chemical profiles of the extracts and their biological activities. Results: Several caffeic acid and flavonoid derivatives were determined in both extracts. The extracts displayed scavenging capacity against the physiologically relevant nitric oxide (^•^NO) and superoxide anion (O_2_^•−^) radicals, significantly reduced NO production in lipopolysaccharide (LPS)-stimulated macrophages (RAW 264.7), and inhibited the activity of hyaluronidase (HAase), acetylcholinesterase (AChE) and butyrylcholinesterase (BChE). Some of these bioactivities were found to be related with the chemical profile of the extracts, namely with 3-caffeoylquinic, 4-caffeoylquinic, chlorogenic, and *p*-coumaric acids, as well as with luteolin and apigenin derivatives. Conclusions: This study reports, for the first time, the potential medicinal properties of aqueous and hydroethanol extracts of camel grass in the RAW 264.7 cell model of inflammation, and in neurologically related conditions.

## 1. Introduction

Since the dawn of humanity, plants have been used in the prophylaxis, relief, and treatment of diseases with several etiological origins. Despite being intrinsically linked to popular wisdom, the medicinal use of plants is abandoning its empiric framework and becoming increasingly supported by scientific evidence [1]. As important sources of new bioactive compounds with promising pharmacological effects, several plant species have been recognized by the World Health Organization for their medicinal properties [2].

Inflammation is one of the main healthcare concerns for which medicinal plants are well-documented [3]. As part of the organism response to cell and tissues injury, inflammation is characterized by the release of several systemic mediators that act together for damage repair. When the cause of inflammation persists, or when the defence mechanisms are deregulated, inflammation can become chronic [4]. Nitric oxide (NO) plays an important role in many physiologic processes, not only as a signalling molecule, but also as neurotransmitter and participant in platelet aggregation inhibition. Under physiologic conditions, NO acts as an anti-inflammatory mediator; however, at higher concentrations, it induces and exacerbates inflammation, leading to tissue damage [5]. This mediator is produced from l-arginine, by the action of a family of nitric oxide synthase (NOS) enzymes. Of them, the inducible nitric oxide synthase (iNOS) isoform, originally expressed in macrophages in response to inflammatory *stimuli*, is crucial in the inflammatory process due to its capacity to increase NO production [5]. Thus, compounds able to inhibit NO overproduction, or to scavenge the reactive species formed during the inflammatory process, are interesting for the resolution of inflammatory frames [4,6].

The appropriate regulation of reactive species is essential for organism homeostasis. Thus, antioxidants play an essential role in the prevention of various pathologies. Reactive nitrogen species (RNS) and reactive oxygen species (ROS) can accumulate, resulting in the oxidation of cellular components and cellular destruction [7]. Apart from its role in inflammation, oxidative stress underlies other pathologies. The progression of neurodegeneration is a classic example; brain structures supporting memory are particularly sensitive to the oxidative status, due to their high demand for oxygen [8]. Most cognitive and behavioural changes are postulated to be caused by deficient brain cholinergic pathways. This can help explain why the enhancement of cholinergic transmission, by extending the availability of acetylcholine (ACh) in the synaptic cleft, can improve the symptoms associated with neurodegeneration. Moreover, the inhibition of acetylcholinesterase (AChE) and butyrylcholinesterase (BChE), enzymes responsible for the hydrolysis of ACh following synaptic release, has thus been suggested as a promising strategy to avoid the progression of dementia [9]. In this regard, diverse medicinal species traditionally used to treat neurological diseases have been evaluated for their cholinesterase inhibitory activity to support their ethnopharmacological applications [9,10].

*Cymbopogon* species have been used in traditional medicine worldwide, lemon grass (*Cymbopogon citratus* (DC.) Stapf) being the most widely distributed and well-studied one [11,12,13]. Camel grass (*Cymbopogon schoenanthus* (L.) Spreng.), on the other hand, has been scarcely explored regarding its chemical profile and pharmacological activities [14,15,16]. Besides the over-studied volatile extracts of *Cymbopogon* spp., identified as essential oils, non-volatile extracts are gaining researchers’ attention for their promising pharmacological applications and lower toxicity [17]. Among the specialized metabolites present in non-volatile extracts of *Cymbopogon* spp., phenolic compounds are likely the most notable ones. In particular, hydroxycinnamic acids and flavonoids can be highlighted for their marked antioxidant and anti-inflammatory properties and capacity to inhibit key enzymes involved in several pathologic processes [18,19,20,21,22].

The aims of this work were to establish the phenolic profiles of aqueous and hydroethanol extracts of camel grass and to evaluate their toxicity, anti-inflammatory and antioxidant potential, together with their effect on enzymes engaged in the neurodegenerative process. In addition to contributing to the enrichment of scientific knowledge on the chemistry of phenols of this still understudied species of the genus *Cymbopogon*, the present work highlights the potential medicinal properties of aqueous and hydroethanol extracts of *C. schoenanthus* in the RAW 264.7 cell model of inflammation, and in neurologically related conditions, for the first time.

## 2. Results and Discussion

The genus *Cymbopogon* is widely known for its high content in essential oils. Though the pharmacological applications of its volatile extracts are well-exploited, other extracts and species from this same genus remain underexplored [12]. Likewise, preliminary *in vitro* data on the potential toxicity of extracts are scarce to non-existent. Therefore, this work focused on the antioxidant, anti-inflammatory and neuromodulating capacity of aqueous and hydroethanol extracts of camel grass, which are among the biological properties most reported in traditional medicine for this genus. Moreover, the toxicity of the extracts was screened in different cell lines, and the effect of the extraction method and solvent composition on their chemical profile and biological activity was also considered. Additionally, a relationship between the biological activities and the phenolic profile was established.

### 2.1. Phenolic Profile

**Figure 1:**
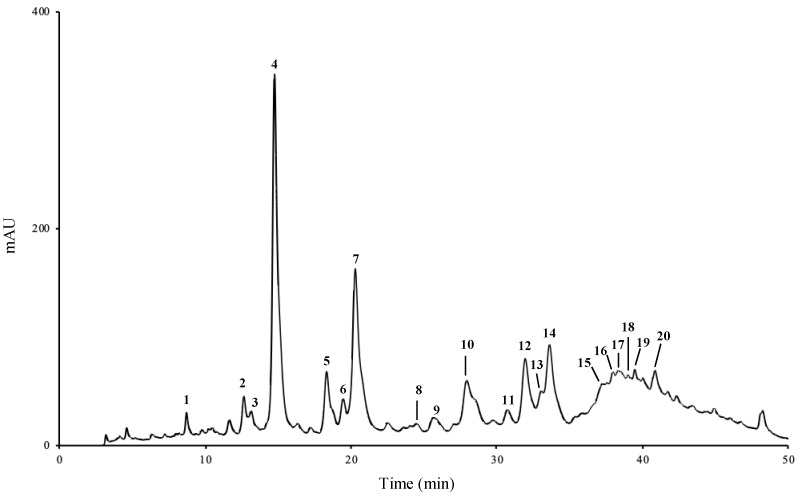
HPLC-DAD chromatogram of camel grass aqueous extract, recorded at 320 nm. **1**: 3-*O*-caffeoylquinic acid; **2**: 4-*O*-caffeoylquinic acid; **4**: chlorogenic acid; ***7***: *p*-coumaric acid; **9**: ferulic acid; **12**: isoorientin; **13**: luteolin glycoside; **14**: luteolin-3′,7-di-*O*-glucoside; **3**, **5**, **6** and **8**: caffeic acid derivatives; **10**, **11**, **15**–**20**: apigenin glycosides.

HPLC-DAD analysis of camel grass extracts revealed similar qualitative compositions, regardless of the extraction solvent and procedure. Figure 1 displays the HPLC-DAD chromatogram obtained with the aqueous extract.

On the other hand, the concentrations of the identified compounds varied according to the nature of the extracting solvent (Table 1). The aqueous extract was significantly richer in phenolic compounds (1.85 mg/g dry plant) than the hydroethanol extract (0.95 mg/g dry plant) (*p* < 0.05). This demonstrates that the infusion process was much more effective in extracting phenolic compounds than the ultrasound-assisted extraction procedure used to obtain the hydroethanol extract (*p* < 0.05). Ultrasound is known for enhancing the rate of mass transfer of analytes to the solvent. Nevertheless, the hydroethanol extracts, obtained with sonication, presented a lower amount of phenols than the extracts obtained by infusion, confirming that the effect of the solvent is more decisive than the extraction method.

Regarding the major subclasses of phenolic compounds determined in camel grass extracts, flavonoid derivatives clearly dominated, when compared to hydroxycinnamic acids, in both aqueous (1.02 vs. 0.83 mg/g of dry plant) and hydroethanol extract (0.62 vs. 0.33 mg/g of dry plant) (Table 1). Among hydroxycinnamic acids, chlorogenic acid (**4**) was the major compound in the aqueous extract, while *p*-coumaric acid (**7**) was the most representative compound in the hydroethanol extract. Among flavonoid derivatives, luteolin-3′,7-di-*O*-glucoside (**14**) was the phenolic found at the highest concentration in both extracts (Table 1).

Studies reporting the chemical profile of camel grass are almost exclusively dedicated to its essential oils. To our knowledge, few studies have reported the phenolic composition of non-volatile extracts of this species. The works by the groups of Khadri [15], Musa [23] and Abu-Serie [24] explored different biological activities of aqueous and methanol (80%) extracts. However, the phenolic profile of the extract was not established, and the total phenolic content was quantified through the non-specific Folin-Ciocalteau colorimetric method. Ben Othman and colleagues [16] determined the phenolic composition of an ethanol extract (70%) of camel grass. In the HPLC-DAD analysis, the authors identified seven phenolic compounds, namely quercetin-3-*O*-rhamnoside, resorcinol, and *trans*-cinnamic, caffeic, 2,5-dihydroxybenzoic, ferulic and gallic acids, the flavonoid being the most representative compound. Najja and co-workers [25] also characterized an ethanol extract (70%) of camel grass by HPLC-DAD, reporting the presence of the same compounds, quercetine-3-*O*-rhamnoside being also the most abundant. Rocchetti and co-workers [26] analysed the phenolic profile of an aqueous extract by triple-TOF mass spectrometry, having found several flavonoids and phenolic acids: kaempferol, quercetin, luteolin and apigenin glycosides were the most representative among the flavonoids, while caffeic, ferulic and coumaric acids predominated among the phenolic acids. These results are in line with those obtained in the study herein. In this work, ferulic acid (**9**) was the only compound that had been previously reported. As far as we are aware, 3-caffeoylquinic (**1**), 4-caffeoylquinic (**2**) and chlorogenic (**4**) acids are being reported here for the first time in camel grass.

Unlike camel grass, lemon grass is, by far, the most well-documented species within the genus. Figueirinha and collaborators [19] determined the composition of lemon grass aqueous extracts, having reported the presence of two classes of phenolic compounds: hydroxycinnamic acids, namely caffeic acid, its derivatives, and *p*-coumaric acid, and flavonoids, mainly 6-C and 8-C glycosyl flavones, derivatives of luteolin and apigenin, which is in accordance with our results (Figure 1, Table 1). As herein, other surveys performed with different extracts from the aerial parts of lemon grass [27] reported chlorogenic acid as the major compound in aqueous extracts, together with two luteolin glycosides, and luteolin derivatives as the most representative of a methanol:water extract. Similar results were later obtained by Campos et al. [28]. More recently, Roriz and colleagues focused on the antioxidant compounds of different plants, having found luteolin derivatives as the major compounds in lemon grass [29,30]. This is in accordance with our findings, in which luteolin glycoside corresponded to ca. 28 and 32% of the total phenolic compounds identified in the aqueous and hydroethanol extracts, respectively.

### 2.2. Antioxidant Activity

**Figure 2:**
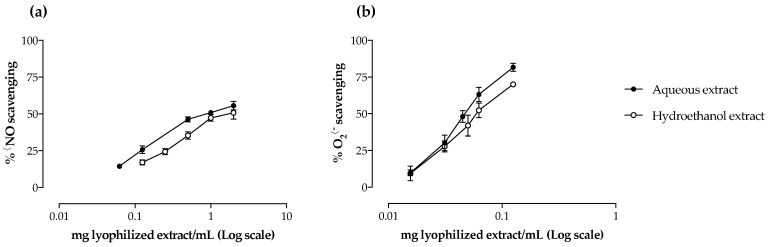
Scavenging effect of camel grass extracts against nitric oxide (^•^NO) (**a**) and superoxide anion (O_2_^•−^) (**b**) radicals generated in a cell-free system. Results are expressed as percentage of the respective control (mean ± SD of at least three determinations, each performed in triplicate).

Camel grass extracts were screened for their antioxidant capacity against ^•^NO and O_2_^•−^. The two extracts were able to scavenge both reactive species in a dose-dependent manner (Figure 2, Table 2). When comparing the IC_50_ values obtained, the aqueous extract was significantly more efficient than the hydroethanol one against ^•^NO (*p* < 0.05), while no significant differences were observed regarding the O_2_^•−^ scavenging ability of both extracts (Table 2). The results obtained for ^•^NO scavenging were less promising than those obtained with the reference standard quercetin (IC_50_ = 58.1 μg/mL); however, regarding O_2_^•-^, the results were quite remarkable, being of the same order of magnitude of the reference standard (IC_50_ = 24.6 μg/mL).

Antioxidants have been implicated in the prevention of various diseases by protecting the organism against cell damage caused by oxidative stress [31]. Although the total amount of phenolic compounds was significantly different between the two extracts tested (*p* < 0.05) (Table 1), their ^•^NO scavenging capacity seemed to rely on the presence of certain compounds. For instance, a negative correlation was observed between the IC_50_ values and 4-caffeoylquinic acid (**2**) (−0.816, *p* < 0.05) and the apigenin glycoside (**15**) (−0.828, *p* < 0.05), while a positive correlation was found for the apigenin glycoside (**16**) (0.889, *p* < 0.05) (Table 3. With regard to O_2_^•−^, data analysis demonstrated that the total amount of phenolic compounds was negatively correlated with the radical scavenging capacity of the extracts (−0.814, *p* < 0.05), mainly due to flavonoids (Table 3).

As far as we know, the evaluation of the antioxidant capacity of camel grass non-volatile extracts was limited to the studies conducted by the groups of Khadri [15] and Rocchetti [26]. The first assessed the effect of an aqueous extract against the non-physiological 1,1-diphenyl-2-picryl-hydrazyl radical (DPPH^•^) and concluded that the antioxidant activity was positively correlated with the phenolic content [15]. The correlation between phenolics and the antioxidant potential of the extracts was corroborated by Rocchetti et al., through the Trolox equivalent antioxidant capacity (TEAC), who also found that flavonoids were strongly correlated with TEAC values [26]. To our knowledge, this is the first work reporting the antioxidant potential of camel grass extracts against free radicals with biological importance (^•^NO and O_2_^•−^) and the first one to establish a relationship between their radical scavenging capacity and their phenolic profile.

Contrary to camel grass, several studies reported the antioxidant potential of non-volatile extracts from species of the same genus, namely lemon grass [19,27]. Cheel and collaborators [27] evaluated the antioxidant potential of several lemon grass extracts, including methanol, methanol:water (7:3 and 1:1, *v*/*v*), infusion and decoction, against DPPH^•^ and O_2_^•−^ and reported a positive correlation between the antioxidant activity and the phenolics content. Our results are in accordance with these, as a higher phenolic content led to lower IC_50_ value (Table 1, Table 2 and Table 3). In another work [19], caffeic and *p*-coumaric acids and apigenin and luteolin derivatives were determined and correlated with the free radical scavenging capacity of the extracts. In accordance with the present study, those authors also suggested that flavonoids, the major subclass of identified compounds, were responsible for the antioxidant potential of the extracts [19].

### 2.3. Anti-Inflammatory Potential

**Figure 3:**
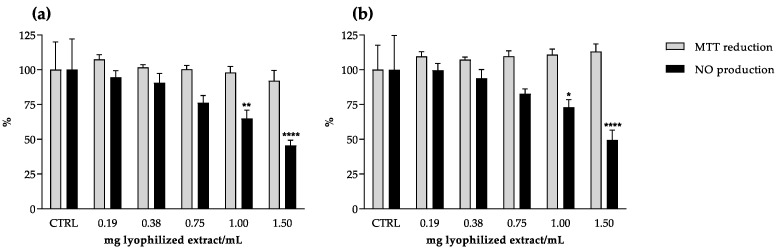
Effect of camel grass aqueous (**a**) and hydroethanol (**b**) extracts on thiazolyl blue tetrazolium bromide (MTT) reduction and nitric oxide (NO) production in lipopolysaccharide (LPS)-stimulated RAW 264.7 cells. Results are expressed as percentage of the respective control (CTRL) (mean ± SD of four determinations, each performed in triplicate). * *p* < 0.05; ** *p* < 0.01; **** *p* < 0.0001 (ANOVA, Tukey HSD multiple comparison test).

The anti-inflammatory potential of camel grass extracts was assessed by a model of macrophages challenged with lipopolysaccharide (LPS). Both extracts were able to reduce cellular NO production in a dose-dependent manner, and no cytotoxicity was observed under the range of concentrations tested (0.19–1.5 mg lyophilized extract/mL) (Figure 3). Despite being less effective than the reference drug dexamethasone (IC_50_ = 34.6 μg/mL), at the highest concentration tested (1.5 mg lyophilized extract/mL), the co-incubation with camel grass extracts reduced NO released by stimulated macrophages by more than 50%, in comparison to the untreated control (Figure 3). However, no correlation was found between the total phenolic content and the reduction of NO released by RAW 264.7 cells.

Inflammation is one of the conditions for which traditional medicine recommends the use of species of *Cymbopogon* genus [12]. To our knowledge, there are no previous reports concerning the anti-inflammatory potential of camel grass in macrophages upon LPS stimulation.

Although no significant differences were found between the IC_50_ values, the extract with higher content of both flavonoids and hydroxycinnamic acids displayed a tendency to be more active (Table 1 and Table 2). This is in accordance with previous studies conducted with macrophages that attributed the anti-inflammatory activity of *Cymbopogon* spp. extracts to their flavonoid content [6,32]. Additionally, even with little expression when compared to essential oils, some authors have explored the anti-inflammatory potential of non-volatile extracts of *Cymbopogon* spp., namely those of lemon grass [18,20,21]. A study conducted with LPS-stimulated RAW 264.7 cells treated with a lemon grass aqueous extract suggested phenolic compounds as the main contributors to the reduction in iNOS expression and NO production [21]. The anti-inflammatory activity of some compounds isolated from lemon grass was further assessed in the same cell model, revealing that luteolin glucosides were partly responsible for the anti-inflammatory properties of the extracts [20]. These observations are in accordance with our results: treatment with the aqueous extract, richer in luteolin glycosides, seemed to have a stronger effect regarding NO reduction in the cell system (Table 1 and Table 2). Another cell model was used to evaluate the anti-inflammatory potential of an infusion of lemon grass, as well as its polyphenol fractions, on the NO produced by LPS-stimulated dendritic cells [18]. The authors demonstrated that the infusion significantly inhibited NO production and iNOS expression; the strongest anti-inflammatory effects were observed for the flavonoid-rich fraction, again indicating luteolin glycosides as the main contributors to the effects. A recent work evaluated the anti-inflammatory capacity of camel grass aqueous and ethanol (50% *v*/*v*) extracts by assessing the inhibition of the active NF-κB pathway in an HT-29 cell line. The authors found that only the aqueous extract was able to significantly inhibit the pro-inflammatory gene expression but did not report a correlation with the phenolic compounds identified [26]. Regarding phenolic acids, Francisco and co-workers reported the contribution of chlorogenic acid to the anti-inflammatory activity displayed by a lemon grass infusion [33]. The authors tested the main phenolic acid in the extract, chlorogenic acid, and found that it maintained the phosphorylation levels of IκBα, as the extract did. The inhibition of p65 translocation to the nucleus by lemon grass extract was also observed, which was consistent with the NF-κB inhibition, suggesting the anti-inflammatory potential of the extract by inhibition of NF-κB activation. Chlorogenic acid is the most representative hydroxycinnamic of the extracts evaluated herein. Consequently, it may also contribute to the anti-inflammatory activity observed in our study.

The mechanisms behind inflammation are complex, accounting for a huge number of mediators and enzymes that may be directly or indirectly involved in the process. High and low molecular weight forms of hyaluronic acid (HA) exhibit opposite effects on cell behaviour. High molecular weight HA inhibits endothelial cell growth, is increased at sites of inflammation, and often correlates with leukocyte adhesion and migration. Studies on activated macrophages have shown that HA fragments induce the expression of chemokine genes, such as macrophage inflammatory proteins (MIP) with a crucial role in initiating and maintaining the inflammatory response [34]. Hyaluronidase (HAase) is an enzyme responsible for the degradation of HA; thus, its inhibition can result in a favourable environment to overcome inflammation.

**Figure 4:**
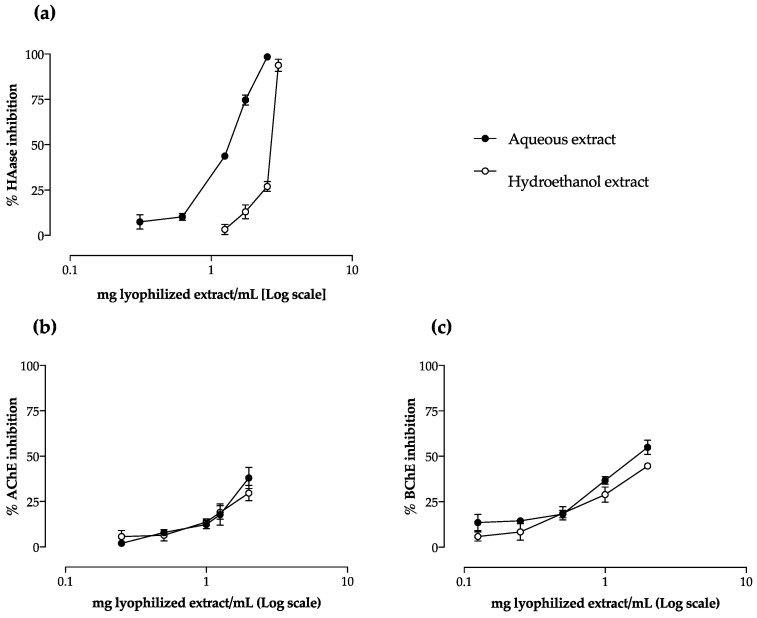
Effect of camel grass extracts on hyaluronidase (HAase) (**a**), acetylcholinesterase (AChE) (**b**), and butyrilcholinesterase (BChE) (**c**) activity. Results are expressed as percentage of control (mean ± SD of, at least, three determinations).

Both extracts demonstrated capacity to inhibit the HAase-mediated degradation of HA in a dose-dependent manner (Figure 4a). At the highest tested concentrations (3.0 and 2.5 mg lyophilized extract/mL for hydroethanol and aqueous extract, respectively), the extracts almost completely inhibited the enzymatic activity (Figure 4a). The aqueous extract was more effective (*p* < 0.05), presenting an IC_50_ value of about half of that obtained for the hydroethanol extract (Table 2), and in the same order of magnitude of the reference drug disodium cromoglicate (1.10 mg/mL).

Regarding HAase inhibition, a clear correlation was observed, not only concerning individual compounds, but also considering the total amount within each subclass (Table S1). Strong negative correlations were found for hydroxycinnamic acids (−0.989, *p* < 0.01) and flavonoids (−0.965, *p* < 0.01), which easily explains the significantly lower IC_50_ value obtained with the aqueous extract (Table 2 and Table 3). To the best of our knowledge, this is the first report devoted to the evaluation of the effect of *Cymbopogon* spp. on HAase.

### 2.4. Effect on AChE and BChE Activity

Camel grass extracts showed a dose-dependent behaviour concerning their capacity to inhibit AChE and BChE (Figure 4b,c). Both extracts displayed a stronger capacity to inhibit BChE than AChE (Table 2): the aqueous extract was the most effective to impair butyrylcholine hydrolysis, being the only one to inhibit half of the enzyme activity (IC_50_ value of 1.76 mg lyophilized extract/mL against BChE).

The neuromodulator properties of *Cymbopogon* spp. have been mostly attributed to their essential oils [35,36]. Adaramoye and Azeez [37] evaluated the capacity of a methanol extract of lemon grass to inhibit AChE, but found no differences compared to the untreated control. Khadri and colleagues [15] evaluated the effect of different camel grass extracts on AChE inhibition and verified that the inhibitory activity increased with the solvent polarity. These previous results are in accordance with our work; even with no statistical differences found, the IC_25_ obtained with the aqueous extract was lower than that of the hydroethanol extract (Table 2). However, both extracts still presented higher IC_25_ values than that of the control drug galantamine (0.86 mg/mL). Regarding BChE, the strongest inhibition was also obtained with the aqueous extract (Table 2), with a promising IC_25_ value about 3 times lower than that of the reference standard galantamine (IC_25_ = 1.81 mg/mL). However, no correlation with the chemical profile was observed, suggesting that this biological activity may result from a synergism between all the compounds. To our knowledge, this is the first work reporting the BChE inhibitory activity of camel grass.

**Figure 5:**
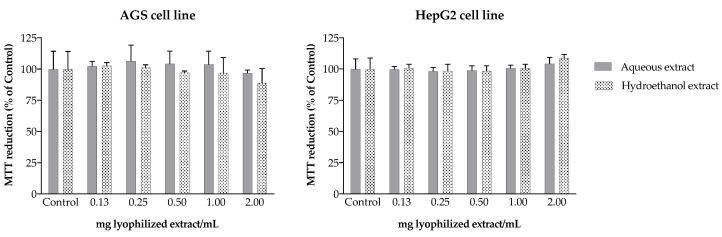
Effect of camel grass extracts on MTT reduction in AGS and HepG2 cells. Results are expressed as percentage of untreated control (mean ± SD of four determinations performed in triplicate).

The disabled concentration of neurotransmitters in the synaptic cleft is associated with the impairment of cognitive functions and the progressive loss of memory. In this way, the consumption of non-volatile extracts of *Cymbopogon* species can be seen as a promising non-pharmacological approach to increase neurotransmitter concentrations, contributing to reduce the adverse symptoms of neurodegenerative diseases. Moreover, the severe side effects associated with the currently commercialized cholinesterase inhibitors, namely related to hepatotoxicity and gastrointestinal disorders [10], may be overcome with inhibitors from natural sources, such as camel grass extracts. In fact, cell viability assays conducted with the human gastric adenocarcinoma (AGS) and liver hepatocellular carcinoma (HepG2) cell lines exposed for 24 h to the different extracts revealed no toxicity under the tested concentrations, relative to the respective control (*p* > 0.05) (Figure 5).

This preliminary *in vitro* toxicological screening encourage the consumption of camel grass extracts under the effective concentrations found in the present work.

## 3. Materials and Methods

### 3.1. Standards and Reagents

Thiazolyl blue tetrazolium bromide (MTT), β-nicotinamide adenine dinucleotide reduced form (NADH), sodium nitroprusside dehydrate (SNP), sulphanilamide, naphtylethylenediamine, ethanol, LPS from *Salmonella enterica*, formic acid, nitrotetrazolium blue chloride (NBT), phenazinemethosulfate (PMS), dimethyl sulfoxide (DMSO), sodium chloride (NaCl), sodium formate, HAase from bovine testes (type IV-S), hyaluronic acid (HA) sodium salt from *Streptococcus equi*, bovine serum albumin (BSA), AChE, acetylthiocholine iodine, BChE, S-butyrylthiocholine chloride, dexamethasone (≥97%), galantamine hydrobromide from *Lycoris* sp. (≥94%) and disodium cromoglycate (DSCG) (≥95%) were purchased from Sigma-Aldrich (St. Louis, MO, USA). Acetic acid (glacial), HPLC-grade methanol, acetonitrile, di-sodium tetraborate and 4-dimethylaminobenzaldehyde (DMAB) were from Merck (Darmstadt, Germany). Dulbecco’s Modified Eagle Medium (DMEM), Dulbecco’s phosphate buffered saline (DPBS), heat-inactivated foetal bovine serum (FBS) and Pen Strep solution (Penicillin 5000 units/mL and Streptomycin 5000 mg/mL) were from Gibco (Invitrogen, Paisley, UK). Murine macrophage-like cell line RAW 264.7, human hepatoma cell line and human gastric adenocarcinoma cell line AGS were from the American Type Culture Collection (LGC Standards S.L.U., Barcelona, Spain). 3-Caffeoylquinic acid (HPLC) ≥ 98%, 4-caffeoylquinic acid (HPLC) ≥ 98%, caffeic acid (HPLC) ≥ 98%, chlorogenic acid (HPLC) ≥ 98%, *p*-coumaric acid (HPLC) ≥ 98%, ferulic acid (HPLC) ≥ 98%, isoorientin (HPLC) ≥ 98%, luteolin-3′,7-di-*O*-glucoside (HPLC) ≥ 98% and vitexin (HPLC) ≥ 98% were purchased from Extrasynthèse (Genay, France). Water was purified using a Milli-Q water purification system (Millipore, Bedford, MA, USA).

### 3.2. Plant Material and Extract Preparation

Camel grass (aerial parts) was obtained from the herbalist “Dermapelle” (São Paulo, Brazil) (www.dermapelle.com.br, accessed on 5 November 2022). Plant identification was attested according to eFloras (2008), and a voucher specimen was deposited at the herbarium of the Laboratory of Pharmacognosy, Faculty of Pharmacy, Porto University. After powdering (particle size < 910 µm), two different extracts were prepared.

*Aqueous extract*: 300 mL of boiling water were added to 3 g of powdered plant material. The mixture was left at room temperature for 15 min, after which the resulting extract was filtered by cotton and subsequently by Buchner funnel. The filtrate was left to cool to room temperature, frozen and kept at −20 °C prior to lyophilization in a Virtis SP Scientific Sentry 2.0 apparatus (Gardiner, NY, USA).

*Hydroethanol extract*: 300 mL of a water:ethanol (1:1 *v*/*v*) mixture were added to 3 g of powdered plant material, and the mixture was subjected to sonication for 30 min. The resulting extract was filtered, following the same procedure used for the aqueous extract. The organic phase was evaporated to dryness under reduced pressure, at 35 °C. The remaining aqueous solution was treated as described above.

The extraction yields were ca. 10.4 and 14.1% for aqueous and hydroethanol extract, respectively. The dried extracts were kept in a desiccator until analyses.

### 3.3. HPLC-DAD Analysis

The analysis was performed on an analytical HPLC unit (Gilson, Lewis Center, OH, USA). Lyophilized extracts were dissolved in ultrapure water (30 mg/mL for the infusion and 20 mg/mL for the hydroethanol extract) and filtered through a 0.45 µm pore membrane. Twenty microliters of the resulting solution were analysed using a Spherisorb ODS2 column (4.6 × 250 mm, 5 µm particle size; Waters, Ireland). A column heater (Column-Thermostat model Jetstream 2 Plus, Hockenheim, Germany) was used to keep temperature at 25 °C during the analyses. The solvent system consisted in methanol (A) and water:formic acid (95:5 *v*/*v*) (B), starting with 5% A and installing a gradient to obtain 15% A at 3 min, 25% A at 22 min, 30% A at 30 min, 45% A at 33 min, 55% A at 38 min, 75% A at 46 min, and 100% A from 48 to 50 min. The solvent flow rate was 0.9 mL/min. Spectral data from peaks were accumulated in the range of 190–600 nm. Data were processed with Clarity chromatography software (DataApex, Prague, Czech Republic). Compounds were identified by comparing their retention times and UV-Vis spectra with those of authentic standards. Phenolic compound quantification was achieved by measuring the absorbance recorded in the chromatograms relative to external standards: hydroxycinnamic acids (**1**–**9**) were determined at 320 nm, and flavonoids (**10**–**20**) at 350 nm.

Caffeic acid derivatives (**3**, **5**, **6**, and **8**) were tentatively identified by comparing their UV spectra with that of caffeic acid, and quantified as caffeic acid; apigenin glycosides (**10**, **11**, **15**–**20**) were tentatively identified by comparing their UV spectra with that of vitexin (apigenin 8-*C*-glucoside), and quantified as vitexin; the luteolin glycoside (**13**) was tentatively identified by comparing its UV spectrum with that of isoorientin (luteolin 6-*C*-glucoside, **12**), and quantified as isoorientin. The other compounds were quantified as themselves. Calibration curves, limit of detection (LOD) and limit of quantification (LOQ) are shown in Table S1.

### 3.4. Superoxide Anion Radical (O_2_^•−^) Scavenging Assay

The anti-radical capacity of lemon grass extracts was evaluated as before [38]. Three independent assays were performed in triplicate. Quercetin was used as positive control.

### 3.5. Nitric Oxide Radical (^•^NO) Scavenging Assay

^•^NO was generated from SNP dehydrate and determined as previously described [39]. Four independent assays were performed in duplicate. Quercetin was used as positive control.

### 3.6. HAase Inhibition Assay

The assay was performed following the protocol proposed by our group [40]. DSCG was used as positive control. Three independent assays were performed in duplicate.

### 3.7. AChE and BChE Inhibition Assays

The capacity to inhibit cholinesterase was determined based on Ellman’s method and following a previously proposed procedure [41]. Galantamine was used as positive control. At least three independent assays were performed in triplicate.

### 3.8. Cell Culture and Treatments

The murine macrophage cell line RAW 264.7, the human hepatoma cell line HepG2 and the human gastric adenocarcinoma cell line AGS were grown at 37 °C, in DMEM supplemented with GlutaMAX™-I, 10% FBS, 100 U/L penicillin and 100 µg/mL streptomycin, in a humidified atmosphere of 5% CO_2_. Cells were inoculated in 96-well plates and cultured until confluence. Camel grass lyophilized extracts were dissolved in DMEM, sterilized by filtration through a 0.22 µm pore membrane and stored at −20 °C until use. Five dilutions of the extracts were prepared in supplemented DMEM immediately before cell exposure. To determine the effect of the extracts on NO production by RAW 264.7 cells, a 2 h pre-treatment with different extract concentrations or vehicle was undertaken, followed by the addition of 1 µg/mL LPS (or vehicle) and a further incubation for 22 h at 37 °C in a humidified atmosphere of 5% CO_2_. The effect on NO production was also evaluated in the absence of LPS, in order to observe possible changes in NO basal levels. No LPS was added to the negative controls. Four independent assays were performed in duplicate.

### 3.9. Toxicity to RAW 264.7 Cells

Cytotoxicity of camel grass extracts was assessed by the MTT assay, as previously described by Barbosa et al. [39]. DMSO (20%) was used as positive control.

### 3.10. NO Release by RAW 264.7 Cells

After the incubation period, the nitrite accumulated in the culture medium was determined using the Griess reaction, as previously reported [39]. Dexamethasone was used as positive control. Four independent experiments were performed in duplicate.

### 3.11. Toxicity to AGS and HepG2 Cells

The human gastric cell line AGS was used as model to assess the camel grass extracts’ gastric toxicity. Cells were seeded at a density of 15,000 cells/well in 96-well plates and, after confluence, incubated with the extracts for 24 h (37 °C). The human hepatoma cell line HepG2 was used to predict the toxicity of the extracts to human liver. Cells were seeded at a density of 10,000 cells/well into 96-well plates and, after confluence, incubated with the extracts for 24 h at 37 °C. The MTT assay was conducted for both cell lines, following the conditions described before. DMSO (20%) was used as positive control.

### 3.12. Statistical Analysis

Statistical analysis was performed using IBM SPSS STATISTICS software, version 24.0, IBM Corporation, New York, NY, USA (2011). Data were analysed for normality and homogeneity of variance by Kolmogorov–Smirnov and Leven’s tests and then submitted to one-way ANOVA, using a Tukey’s HSD (honest significant difference) as post hoc test for cell assays, or to a two-tailed unpaired t-test to compare the total content of phenolic compounds and the IC_50_ values of the bioactivity assays. IC_50_ values (expressed in mg of lyophilized extract/mL), concerning both cell-free and cell assays were presented as mean ± SD of at least three independent experiments. A Pearson correlation test was used to compare normalized expression data between the chemical profile and the biological activities of camel grass extracts.

## 4. Conclusions

The biological activities of camel grass extracts explored herein provided evidence that supports the use of this species in traditional medicine, encouraging its consumption as a non-pharmacological measure for the treatment and relief of inflammation and neurodegeneration-associated conditions. Some of these bioactivities are intimately related with the chemical profile. In fact, the consumption of aqueous extract seems to be advantageous compared to the hydroethanol one, as it is effective in lower doses and can be directly consumed without the need for technological manipulation. As demonstrated herein, camel grass constitutes a natural source of compounds with promising antioxidant and anti-inflammatory potential that can act in several mediators and enzymes related to the inflammatory process. Due to their capacity to inhibit key enzymes involved in the process of neurodegeneration, bioactive extracts can also be promising as a natural alternative to synthetic neuromodulators that influence mood and memory, and their incorporation in new functional foods may create new added-value products.

## Figures and Tables

**Table 1:** Quantification of the phenolic compounds identified in camel grass extracts ^1^.

Compounds		*R*t (min)	Aqueous Extract	Hydroethanol Extract
	**Hydroxycinnamic acids**			
**1**	3-Caffeoylquinic acid	8.66	0.03 ± < 0.01	0.08 ± < 0.01
**2**	4-Caffeoylquinic acid	12.57	0.04 ± < 0.01	0.01 ± < 0.01
**3**	Caffeic acid derivative	13.09	0.02 ± < 0.01	0.01 ± < 0.01
**4**	Chlorogenic acid	14.66	0.49 ± 0.04	0.02 ± 0.01
**5**	Caffeic acid derivative	18.22	0.04 ± > 0.01	nq
**6**	Caffeic acid derivative	19.33	0.02 ± > 0.01	nq
**7**	*p*-Coumaric acid	20.21	0.18 ± 0.01	0.16 ± < 0.01
**8**	Caffeic acid derivative	24.45	0.00 ± < 0.01	nq
**9**	Ferulic acid	25.51	0.01 ± < 0.01	0.05 ± 0.01
	**Σ**		**0.83 ^a^ ± 0.05**	**0.33 ^b^ ± 0.02**
	**Flavonoids**			
**10**	Apigenin glycoside	28.50	0.06 ± < 0.01	0.03 ± < 0.01
**11**	Apigenin glycoside	30.60	0.01 ± < 0.01	nq
**12**	Isoorientin	31.81	0.13 ± 0.01	0.08 ± 0.01
**13**	Luteolin glycoside	32.89	0.07 ± 0.01	nq
**14**	Luteolin-3′,7-di-*O*-glucoside	33.46	0.31 ± 0.03	0.22 ± 0.01
**15**	Apigenin glycoside	37.10	0.09 ± 0.01	0.03 ± 0.02
**16**	Apigenin glycoside	37.91	0.03 ± < 0.01	0.06 ± 0.01
**17**	Apigenin glycoside	38.27	0.07 ± 0.01	0.06 ± 0.01
**18**	Apigenin glycoside	38.96	0.09 ± 0.01	0.04 ± 0.01
**19**	Apigenin glycoside	39.90	0.05 ± 0.01	0.03 ± 0.01
**20**	Apigenin glycoside	40.73	0.11 ± 0.01	0.07 ± 0.01
**Σ**			**1.02 ^a^ ± 0.1**	**0.62 ^b^ ± 0.09**
**Total**			**1.85 ^a^ ± 0.15**	**0.95 ^b^ ± 0.11**

^1^ Values are expressed in mg/g of dry plant, as mean ± SD of four determinations; nq—not quantified. Different superscript letters in each row indicate statistical differences at *p* < 0.05 (two-tailed unpaired *t*-test).

**Table 2:** IC_50_ values (mg lyophilized extract/mL) obtained for the antioxidant, anti-inflammatory and enzyme inhibitory capacity of camel grass extracts ^1^.

	Aqueous Extract	Hydroethanol Extract
^•^NO scavenging	0.93 ± 0.19 ^a^	1.27 ± 0.20 ^b^
O_2_^•−^ scavenging	0.05 ± < 0.01	0.06 ± 0.01
NO reduction in RAW 264.7 cells	1.32 ± 0.17	1.38 ± 0.04
HAase	1.40 ± 0.07 ^a^	2.57 ± 0.17 ^b^
AChE^ 2^	1.49 ± 0.17	1.69 ± 0.21
BChE^2^	0.68 ± 0.02 ^a^	0.82 ± 0.12 ^b^

^1^ Values are expressed as mean ± SD of at least three independent determinations, each performed in triplicate. Different superscript letters in each row indicate statistical differences at *p* < 0.05 (two-tailed unpaired *t*-test). ^2^ Values correspond to 25% inhibition. AChE, acetylcholinesterase; BChE, butyrylcholinesterase; HAase, hyaluronidase; ^•^NO, nitric oxide radical; NO, nitric oxide, O_2_^•−^, superoxide anion radical.

**Table 3:** Correlation between the biological activities and the chemical profile of camel grass extracts ^1,2^.

	Compounds	Antioxidant Activity		Enzyme Inhibition	
		^•^NO Scavenging	O_2_^•−^ Scavenging	HAase	AChE
	**Hydroxycinnamic acids**				
**1**	3-Caffeoylquinic acid			0.981 **	
**2**	4-Caffeoylquinic acid	−0.816 *		−0.944 **	
**3**	Caffeic acid derivative		−0.872 *	−0.827 *	
**4**	Chlorogenic acid			−0.978 **	
**5**	Caffeic acid derivative			−0.974 **	
**6**	Caffeic acid derivative			−0.952 **	
**8**	Caffeic acid derivative				−0.998 *
**9**	Ferulic acid		0.904 *	0.961 **	
	**Σ**			−0.989 **	
	**Flavonoids**				
**10**	Apigenin glycoside		−0.888 *	0.965 **	
**12**	Isoorientin			−0.932 **	
**13**	Luteolin glycoside				−0.998 *
**14**	Luteolin-3′,7-di-*O*-glucoside			−0.864 *	
**15**	Apigenin glycoside	−0.828 *		−0.898 **	
**16**	Apigenin glycoside	0.889 *		0.896 **	
**17**	Apigenin glycoside		−0.928 **		
**18**	Apigenin glycoside		−0.871 **	−0.930 **	
**19**	Apigenin glycoside		−0.940 **	−0.896 **	
**20**	Apigenin glycoside		−0.941 *	−0.868 *	
	**Σ**			−0.965 **	
	**Total**		−0.814 *	−0.974 **	

^1^ Significance levels set at * p < 0.05 and ** p < 0.01. ^2 •^NO, nitric oxide radical; O_2_^•−^, superoxide anion radical; HAase, hyaluronidase; AChE, acetylcholinesterase.

## Data Availability

Not applicable.

## Supplementary Materials

The following supporting information can be downloaded at: https://www.mdpi.com/article/10.3390/molecules27227707/s1, Table S1: Calibration curves of authentic standards used for quantification of different phenolic compounds.
