# Supplementary material for: Camel Grass Phenolic Compounds: Targeting Inflammation and Neurologically Related Conditions
Source: Molecules. 2022 Nov 9;27(22):7707. doi: 10.3390/molecules27227707 (PMC9694793; doi:10.3390/molecules27227707)
Supplement: Supplementary file 1 [file molecules-27-07707-s001.zip › molecules-1996243-supplementary.pdf]

Table S1. Calibration curves of authentic standards used for quantification of different phenolic compounds.

Standards	Calibration curve	r^2	LOD (mg/mL) [a]	LOQ (mg/mL) [b]
3-Caffeoylquinic acid (**1**)	y = 69518x – 47.24	0.9998	0.000095	0.000318
4-Caffeoylquinic acid (**2**)	y = 64021x + 34.62	0.9996	0.007381	0.024602
Caffeic acid	y = 136046x + 102.37	0.9992	0.000078	0.000259
Chlorogenic acid (**4**)	y = 57186x + 844.05	0.9950	0.000571	0.001905
p-Coumaric acid (**7**)	y = 111714x – 358.62	0.9987	0.000867	0.002889
Ferulic acid (**9**)	y = 136245x + 75.344	0.9996	0.000488	0.001627
Isoorientin (**12**)	y = 104640x – 350.48	0.9965	0.000689	0.002295
Luteolin-3′,7-di-*O*-glycoside (**14**)	y = 49540x + 228.19	0.9990	0.001162	0.003875
Vitexin	y = 51676x + 343.74	0.9974	0.000916	0.003054

[a]LOD: limit of detection. [b] LOQ: limit of quantification.
